# BAP1 complexes with YY1 and RBBP7 and its downstream targets in ccRCC cells

**DOI:** 10.1515/biol-2025-1140

**Published:** 2025-07-18

**Authors:** Ying Wu, Xue-Ying Li, Jin-Dong Chen, You-Fu Pan

**Affiliations:** Department of Medical Genetics, Zunyi Medical University School of Preclinical Medicine, 6 Xuefuxi Rd., Xinpu District, Zunyi, Guizhou, 563003, China; Exploring health LLC., Guangzhou, Guangdong, 510663, China; Key Laboratory of Gene Detection and Treatment in Guizhou Province, Zunyi, Guizhou, 563003, China

**Keywords:** *BAP1*, *YY1*, *RBBP7*, ccRCC, kidney cancer, renal cell carcinoma

## Abstract

Clear cell renal cell carcinoma (ccRCC) is the most common type of kidney cancer. A few genes, such as *BAP1*, are associated with the tumorigenesis of ccRCC. Mutations in *BAP1* are related to a proportion of ccRCCs. In this study, to explore the functional role of *BAP1* in ccRCC, the potential interacting proteins with *BAP1* in ccRCC cells are to be identified, and the gene expression profiles of *BAP1* knockdown 786-O cells are to be analyzed.

## 1 Introduction

Renal cell carcinoma (RCC) is a heterogeneous group of cancers derived from renal tubular epithelial cells, accounting for 5% of all malignant tumors in males [1]. Major RCC subtypes include clear cell renal cell carcinoma (ccRCC), papillary RCC, and chromophobe RCC [2]. There are approximately 209,000 new cases per year, and about 102,000 people die from this disease [3]. About 2–3% of cases are familial, and several RCC-related genes have been described, including *VHL, PBRM1, TSC1/TSC2, FLCN, BAP1, FH, STED2, JARID1c/KDM5C,* and *MTOR* [4,5,6,7,8]. Of them, *VHL*, *PBRM1, SETD2*, and *BAP1* are located on chromosome 3p, and mutations in these genes lead to ccRCC. ccRCC caused by *VHL* mutation accounts for >50% [9], while *PBRM1* mutation accounts for 40%, *SETD2* for 16%, and *BAP1* for 15% [4]. The *BAP1* gene, encoding a protein of 729 amino acids called BRCA1-associated protein 1 [10], is a nuclear ubiquitin carboxy-terminal hydrolase. BAP1 regulates a number of biological processes, including DNA damage repair, cell cycle control, chromatin modification, programmed cell death, and the immune response [10,11,12]. Mutations in the *BAP1* gene lead to several aggressive cancers, including RCC, malignant mesothelioma, uveal melanoma, and cutaneous melanoma. Several studies have reported that BAP1 is associated with the occurrence and progression of clear cell RCC [4,13,14]. While a single knockout of *Vhl* and *Pbrm1* failed to develop kidney cancer in mice, both *Bap1-Vhl* and *Bap1-Pbrm1* double-knockout mice developed ccRCC [15,16,17]. Although numerous studies have revealed that *BAP1* is involved in the occurrence of ccRCC [4,13,14], the mechanism leading to *BAP1*-mutated tumorigenesis of ccRCC is unclear. This study aimed to determine the role of BAP1 in ccRCC by exploring the BAP1 interactive proteins and investigate the pathogenesis of ccRCC with *BAP1* mutations.

## 2 Materials and methods

### 2.1 Cell line

The human kidney cell line, 786-O, was purchased from Cell Bank of Chinese Science Academy and was cultured in RPMI-1640 medium (cat. no. 61870044; Gibco; Thermo Fisher Scientific, Inc.) supplemented with 10% fetal bovine serum (cat. no. 11011-6125; Zhejiang Tianhang Biotechnology Co., Ltd.), 2 g/l NaHCO_3_, 100 µg/ml streptomycin, and 100 U/ml penicillin (BBI Life Sciences) at 37°C in a humidified incubator with 5% CO_2_.

### 2.2 Co-immunoprecipitation

786-O cells (3 × 10^5^) were seeded in six-well-plate and incubated at 37°C for 24 h. Cells were lysed with 500 μl of immunoprecipitation (IP) buffer (0.303 g Tris, 0.8775 g NaCl, 0.029 g ethylenediamine-tetraacetic acid, 1 g NP-40, and 5 ml glycerin in 100 mL ddH_2_O, pH 7.4) and followed by ultrasonic treatment for 1 min with a sonicator (SCIENTZ-IID, Ningbo, Zhejiang). Then, the cell lysate was centrifuged at 13,000*g* for 10 min (iCEN-24R, Allsheng, Hangzhou, China). Subsequently, the supernatant was collected and transferred into a new 1.5 ml Eppendorf tube. Approximately 100 μl supernatant was used for input control, and the left was evenly divided into two parts for IgG and BAP1 treatment, respectively. Then, pre-treated Rec-protein A-Sepharose^®^ 4B (Invitrogen) was added into the lysate of IgG and BAP1 groups and mixed with a rotary shaker at 4℃ for 3 h, followed by centrifuging at 4℃, and supernatants were transferred into new 1.5 ml Eppendorf tubes. Then, 2 μg normal mouse IgG and 20 μl Rec-protein A-Sepharose^®^ 4B were added into the IgG tube, followed by 2 μg mouse-anti-human BAP1cross-linked-agarose monoclonal antibody (25% agarose) (Santa CruzBiotechnology), then incubated at a rotary shaker at 4℃ overnight [4]. The mixtures were centrifuged shortly and washed three times with TBS buffer (0.6057 g Tris-HCl and 0.8775 g NaCl in 100 mL ddH_2_O, pH 7.4) at 4℃ for 10 min each time. Loading buffer was added to the pellets and boiled. The mixtures were shortly spin, and the supernatants were subjected to Western blot analysis.

### 2.3 Knockdown of BAP1 in 786-O cell line

786-O cells at 75–80% confluence were transfected with BAP1-short hairpin RNA (shRNA) or control shRNA (Santa Cruz Biotechnology), and polybrene was used to improve the transfecting rate. Puromycin was used to select and stabilize the BAP1-shRNA transfected cells after 12 h of transfection. After 48 h, cells were washed three times with PBS and lysed with IP buffer on ice. The cell lysate was then collected in a 1.5 ml Eppendorf tube and centrifuged at 12,000*g* at 4°C for 10 min. The resultant supernatants were transferred into new 1.5 ml Eppendorf tubes, and protein concentration was determined using bicinchoninic acid assay (cat. no. PC0020; Beijing Solarbio Science & Technology Co., Ltd.) in accordance with the manufacturer’s protocol. The protein was stored at −80˚C for following Western blot analysis.

### 2.4 Western blot

Protein (∼20 μg) was mixed with 4X loading buffer and denatured by boiling for 5 min, after which samples were separated using 11% sodium dodecyl sulfate polyacrylamide gel electrophoresis. Proteins were subsequently transferred to polyvinylidene fluoride membranes (0.22 μm) and blocked with 5% non-fat milk in tris - buffered saline Tween (TBST) (with 0.1% Tween) for 2.5 h at room temperature. Samples were then incubated with primary antibodies BAP1 (28383, Santa Cruz Biotechnology) and glyceraldehyde-3-phosphate dehydrogenase (GAPDH) (ProteinTech Group) at 4°C overnight. The membrane was washed three times with TBST buffer. Goat anti-mouse secondary antibodies (1:1,000, ProteinTech Group) were then added and incubated at 37°C for 2 h. Protein was exposed with enhanced chemilucinescent substrate reagents (cat. no. WBKLS0050; Merck Millipore) following the manufacturer’s protocol. Gel imaging was performed with the Gel Doc XR + System (Gel Doc XR, Bio-Rad), and IPP software (version number. 6.0; Media Cybernetics Inc.) was adopted to measure the gray level of each resultant band.

### 2.5 Quantitative real-time reverse transcriptase polymerase chain reaction (qRT-PCR)

786-O cells were transfected with BAP1-shRNA or control shRNA, as aforementioned. Total RNA was extracted by using TRIzol reagent following the manufacturer’s protocol. Approximately 1 μg total RNA was transcribed into cDNA using PrimeScript™ RT reagent kit (Perfect Real Time) in accordance with the manufacturer’s protocol. For qRT-PCR, 1 μl diluted (1:10 dilution in ddH_2_O) cDNA was mixed with BAP1 primers (20 pM each), and GAPDH was used as an internal control in 10 μl total reaction volume. qRT-PCR was performed using the SYBR^®^ Premix Ex TaqTMII (Tli RNaseH Plus) kit (TaKaRa Biotechnology) according to the manufacturer’s instructions. *BAP1* primers: *BAP*1-FW, CCACCAGCTGATACCCAACTC; *BAP*1-RV, CCACGCTGCTGCAGTTCA. *GAPDH* primers: *GAPDH*-FW, AAGCTAGTTACAAAAAGGCCATCATT; *GAPDH*-RV, AGGGTTCGGACTCCTGGAA.

For differential gene expression analysis, the primers used for qRT-PCR are as follows: *BAP*1-FW, CCACCAGCTGATACCCAACTC; *BAP*1-RV, CCACGCTGCTGCAGTTCA; *GAPDH*-FW, AAGCTAGTTACAAAAAGGCCATCATT; *GAPDH*-RV, AGGGTTCGGACTCCTGGAA; *E2F*8-FW, TGGTCAGATCAGGCTTCGTT; *E2F*8-RV, CTTGCTTTGTACCGGCTGTT; *FGF*21-FW, GGTCGGATGGAGGAGAAACT; *FGF*21-RV, CAAAGTGGAGCTAGGGGACA; *HIST1H1B*-FW, CTTCACTGCCTTTTTCGCCC; *HIST1H1B*-RV, TTAAGCTGGGCCTCAAGAGC; *KLF*15-FW, GCAGCCATTTGAAACCCTGA; *KLF*15-RV, TCCCAGTTGGCCCATTATGT; *HSPA*6-FW, TTGAACTCAGTGGCATCCCT; *HSPA*6-RV, CCTTACCTGTGCTCCTGTCA; *IL1B*-FW, TCCAGCTACGAATCTCCGAC; *IL1B*-RV, AGGTGCTCAGGTCATTCTCC. All the primers were purchased from Sangon Biotech (Shanghai, China).

### 2.6 Cytometry

786-O cells (∼3 × 10^5^) were seeded in each well of a six-well-plate and incubated at 37°C for 24 h. Cells were then treated with BAP1-shRNA lentivirus and control shRNA lentivirus. Cells were counted between 0 and 4 days by cytometry. Growth curves were then generated to assess the effect of BAP1 knockdown on cell growth.

### 2.7 Gene expression profiling

786-O cells transfected with BAP1-shRNA lentivirus and control shRNA lentivirus, as aforementioned, were collected for gene expression profiling. Gene expression profiling was carried out by BGI Genomics (Shenzhen, China) through the BGISEQ-500 platform. Experiments with BAP1 shRNA and control shRNA were performed in triplicate.

### 2.8 Statistics

All data were shown as *X̅* ± *s*. GraphPad Prism was adopted to analyze the data. Independent samples *t*-test was used to detect sample differences. *P* < 0.05, *P* < 0.01, and *P* < 0.001 mean statistically significant, very significant, and extremely significant, respectively.

## 3 Results

### 3.1 Identification of BAP1-interactive proteins through co-immunoprecipitation

**Figure 1:**
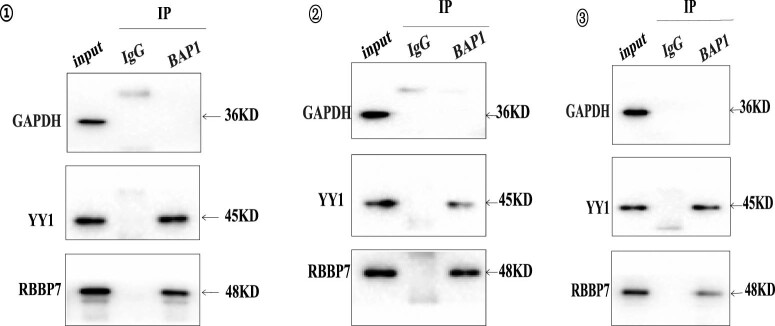
Identification of BAP1 interactive proteins in 786-O cells by co-immunoprecipitation. With anti-GAPDH antibody, only the input lane showed a GAPDH band. With anti-YY1 and anti-RBBP7 antibodies, lanes input and BAP1 presented YY1 and RBBP7 bands. The co-immunoprecipitation experiment was repeated three times.

Previous studies have revealed that BAP1 is able to interact with YY1 and RBBP7 [18,19]. To further determine whether YY1 and RBBP7 are BAP1-binding proteins in ccRCC, we have performed co-immunoprecipitation with YY1 and RBBP7 antibodies (Figure 1). Our results indicated that YY1 and RBBP7 were detected in the BAP1 group. The co-immunoprecipitation was repeated three times, and similar results were obtained (Figure 1). These results demonstrated that BAP1 could bind YY1 and RBBP7.

### 3.2 Knockdown of BAP1 inhibits cell proliferation

**Figure 2:**
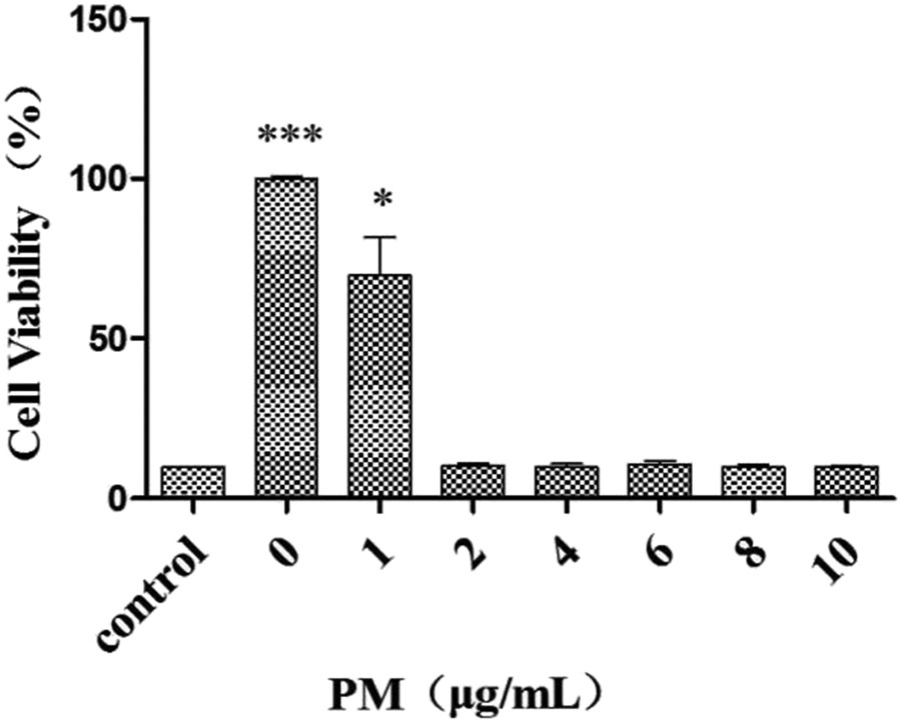
Killing effect of different concentrations of puromycin on 786-O cells. Note: *P < 0.05; ***P < 0.001.

**Figure 3:**
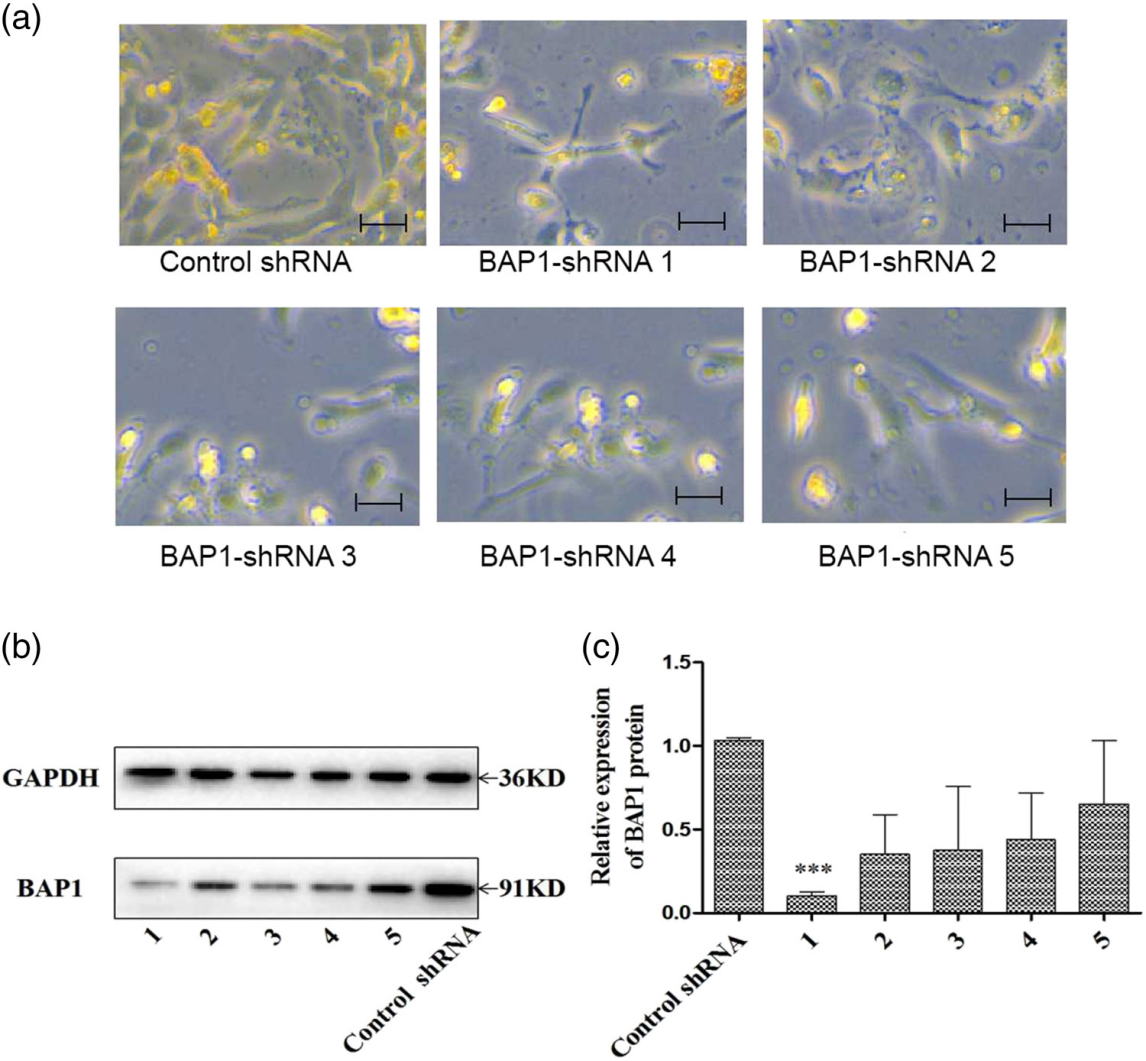
Selection of BAP1-shRNA knockdown 786-O cell clones. (a) BAP1-shRNA transfected cells were selected with 2 μg/ml of puromycin. Five clones (1–5) were taken for BAP1 expression analysis. (b) Representative images for five BAP1-shRNA knockdowns and control; (c) quantitative BAP1 expression of five BAP1-shRNA clones and control shRNA cells. Clone 1 showed the best knockdown result. Scale bar: 100 μm, ***P < 0.001.

Puromycin (2 μg/ml) was used to select the shBAP1-transfected cells for 96 h after transfection (Figures 2 and 3).

Compared to the control group, BAP1-shRNA knockdown cells presented low BAP1 expression at RNA and protein levels. Five shBAP1 knockdown 786-O cell clones were selected for analysis. Of them, Clone A presented the best knockdown effect (0.036 ± 0.108, *P* < 0.001) compared to control and was used for the following experiments (Figure 3).

In addition, BAP1 shRNA clone 1 grows much slower compared to the control shRNA clone. This result indicated that knockdown of BAP1 led to the growth inhibition of 786-O cells.

## 4 Knockdown of BAP1 leads to down-regulation of *E2F8*, *FGF*21, *HIST1H1B* in 786-O cells

### 4.1 Differential expressed genes

**Figure 4:**
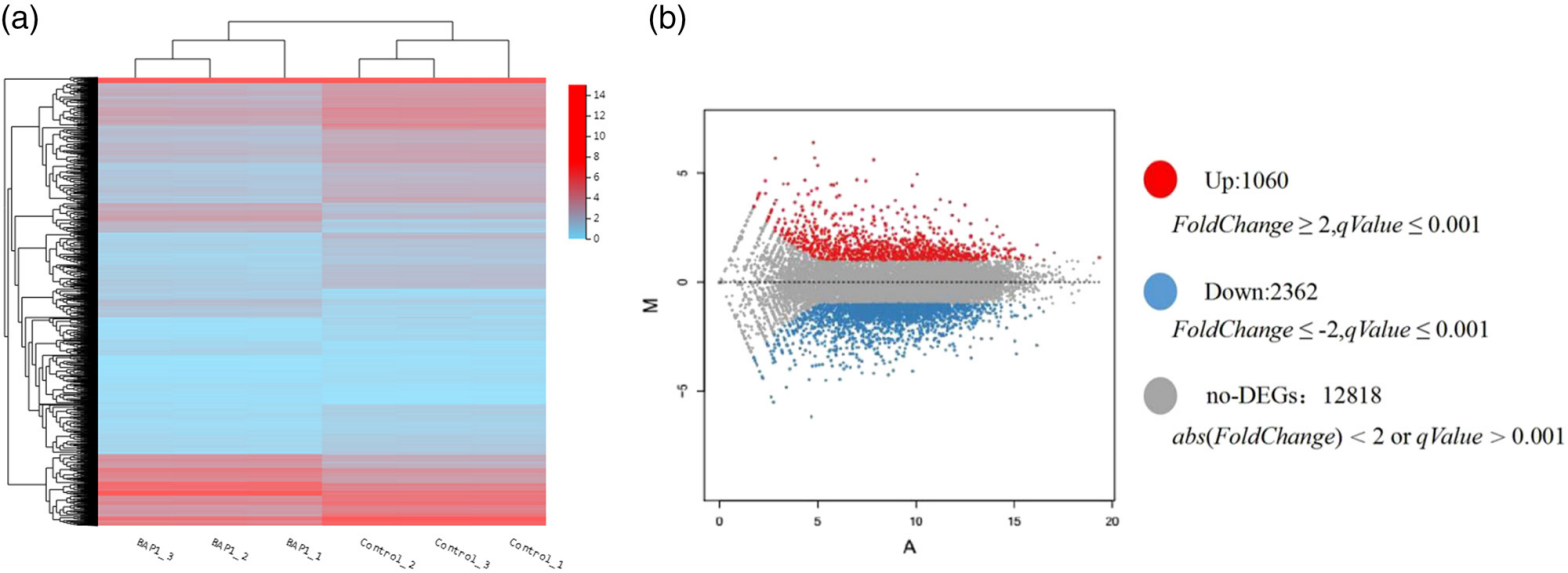
Analysis of differential gene expression. (a) Heatmap of differential expression cluster. *X*-axis, log2 (FPKM+) of the sample, *Y*-axis, differential genes. Red, high expressional genes; Blue, low expressional genes. (b) MA-plot distribution map of DEG from BAP1-shRNA cells. *X*-axis, *A* value (mean of expression log2); *Y*-axis, *M* value (differential folds of expression log2). Red, DEG up-regulated; blue, DEG down-regulated; gray, non-DEG.

We tested 17,202 genes by comparing the BAP1 shRNA group and the control shRNA group and identified 3,422 genes with differential expression. Of them, 1,061 genes were up-regulated and 2,362 down-regulated (Figure 4).

### 4.2 Enrichment of differential expressed genes

**Figure 5:**
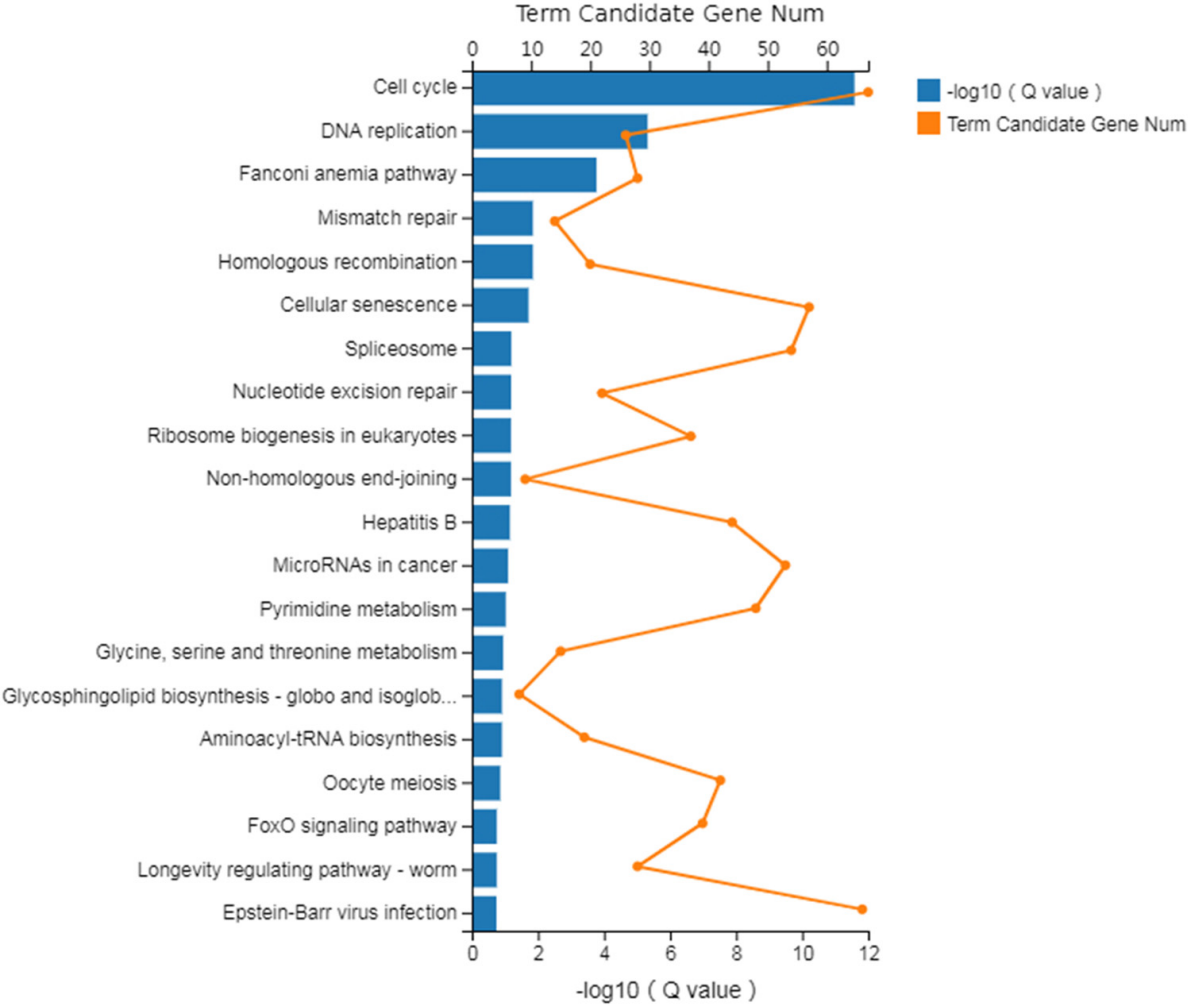
Cluster map of KEGG pathways of differential expression genes identified in 786-O cells with BAP1-shRNA knockdown. *Y*-axis, pathway; down *X*-axis, blue bar, *Q* value (−log10); up *X*-axis, orange line, number of differential genes.

Further analysis indicated that gene cluster/enrichment is associated with 336 pathways in BAP1 shRNA 786-O cells. Of them, the first enriched genes are presented in Figure 5. Sixty-seven gene enrichments are related to the cell cycle, which is associated with Epstein–Barr virus infection, cellular senescence, and spliceosome (Table 1).

**Table 1:** Number of differential genes and related pathways identified in BAP1-shRNA cells

Pathway	Number of genes	*P*-value	Confidence level
Cell cycle	67	7.69 × 10^−15^	<0.01
DNA replication	26	2.84 × 10^−8^	<0.01
Fanconi anemia pathway	28	1.50 × 10^−6^	<0.01
Mismatch repair	14	0.000180521	<0.01
Homologous recombination	20	0.000210901	<0.01
Cellular senescence	57	0.000344742	<0.01
Spliceosome	54	0.001329485	<0.01
Nucleotide excision repair	22	0.001543348	<0.01
Ribosome biogenesis in eukaryotes	37	0.001784738	<0.01
Non-homologous end-joining	9	0.001952161	<0.01
Hepatitis B	44	0.00230733	<0.01
MicroRNAs in cancer	53	0.002859731	<0.01
Pyrimidine metabolism	48	0.003637364	<0.01
Glycine, serine, and threonine metabolism	15	0.004692524	<0.01
Glycosphingolipid biosynthesis – globo and isoglobo series	8	0.005580328	<0.01
Aminoacyl-tRNA biosynthesis	19	0.005839234	<0.01
Oocyte meiosis	42	0.006998931	<0.01
FoxO signaling pathway	39	0.009724549	<0.01
Longevity regulating pathway – worm	28	0.009960035	<0.01
Epstein–Barr virus infection	66	0.01073498	<0.05

### 4.3 Validation of differential genes

Six differentially expressed genes, *E2F*8, *FGF*21, *HIST1H1B*, *KLF*15, *HSPA*6, and *IL1B*, were selected for further validation by qRT-PCR. Total RNAs were extracted from BAP1-shRNA knockdown 786-O cells and were subsequently used for qRT-PCR analysis.

**Figure 6:**
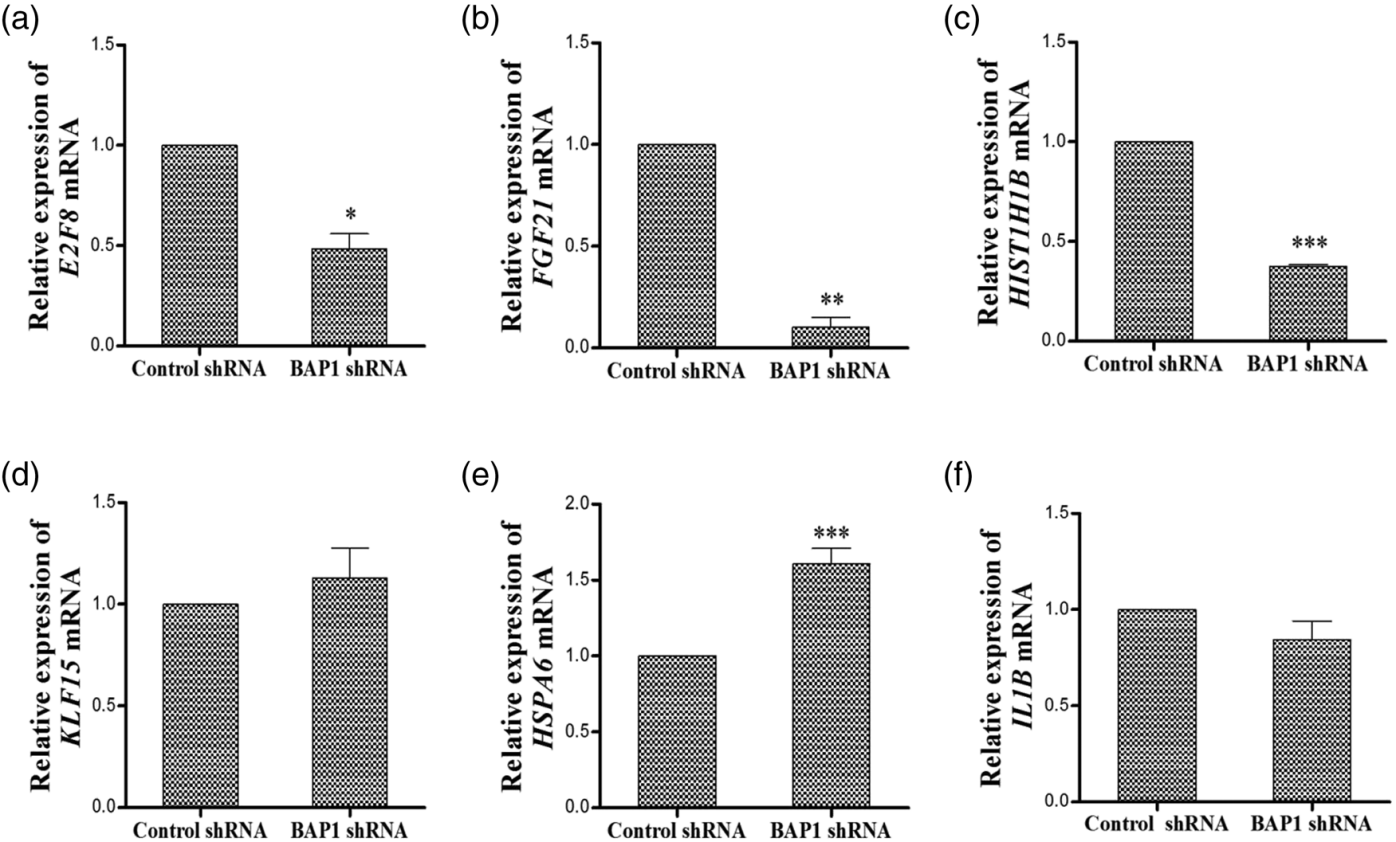
Validation of differential genes in BAP1-shRNA treated cells. At the mRNA level, *E2F*8, *FGF*21, and *HIST1H1B* were down-regulated. *HSPA*6 was up-regulated, while *IL1B* and *KLF*15 had no change. (a) *E2F*8; (b) *FGF*21; (c) *HIST1H1B*; (d) *KLF*15; (e) *HSPA*6; (f) *IL1B*.

*E2F8*, *FGF*21, and *HIST1H1B* were down-regulated when BAP1 was knocked down (Table 2, Figure 6), while *HSPA*6 was up-regulated. We failed to validate the expression change of *KLF*15 and *IL1B* by qRT-PCR at the RNA level, though up-regulation was observed in differential gene expression analysis (Figure 6, Table 3).

**Table 2:** Expressional levels of selected differential expression genes in BAP1-shRNA cells

Gene	mRNA expression (*X* ± *s*)
*E2F*8	0.4839 ± 0.1066 (*P <* 0.05)
FGF21	0.1004 ± 0.0693 (*P <* 0.01)
*HIST1H1B*	0.3761 ± 0.0106 (*P <* 0.001)
*KLF*15	1.1293 ± 0.2079
*HSPA*6	1.7543 ± 0.2316 (*P <* 0.001)
*IL1B*	0.8740 ± 0.1018

**Table 3:** Expressional comparison of differential genes in differential gene expression analysis and qRT-PCR

Gene	Folds of differential expression	qRT-PCR
*E2F*8	−4.46194091289301	Down
*FGF*21	−4.80986421631332	Down
*HIST1H1B*	−5.00250929425571	Down
*KLF*15	6.1674157071866	—
*HSPA*6	5.59243729503806	Up
*IL1B*	4.63473659739819	—

## 5 Discussion

BAP1, a tumor suppressor [11,12], utilizes its deubiquitinating activity to modulate a number of processes, including DNA damage repair, cell cycle control, programmed cell death, cellular differentiation, cell proliferation, chromatin modification, and the immune response. BAP1 is commonly mutated in ccRCC [4,13,14]. However, its role in tumorigenesis of ccRCC is poorly understood. To date, the treatment options available for tumors with BAP1 mutations are limited to standard therapies. Thus, investigation of the role of BAP1 in ccRCC tumorigenesis is essential for the development of target therapy for BAP1-mutated ccRCC. In this study, we demonstrated that BAP1 may inhibit renal tumorigenesis by interacting with YY1, RBBP7, HCF-1, H2A, ASXL1/2, and FoxK1/K2, which is consistent with previous reports [18,19,20,21].

YY1 is a multifunctional transcription factor that plays a critical role in regulating the expression of the genes involved in various physiological processes, including development, cell proliferation, differentiation, DNA repair, and apoptosis. YY1 has emerged as a promising target for antitumor therapy in recent years due to its critical role in regulating various hallmarks of cancer, such as tumor cell proliferation, evading programmed cell death, deregulated metabolism, induction of angiogenesis, activation of invasion and metastasis, genome instability, and evading immune system [11,19,20,21], as well as in tumor cell drug resistance [22,23,24]. Overexpression of YY1 is frequently observed in various human cancers, including breast, bladder, cervical, colon, esophageal, liver, brain, and gastric cancers [23]. While YY1 promotes tumor growth by stabilizing HIF-1α levels in some types of tumors [24], aberrant expression of YY1 results in ccRCC by increasing the expression of HIF-2α and inhibiting VHL [25]. YY1 is able to regulate gene expression by binding BAP1-HCF1 and forming a triplex [26]. In this study, we demonstrated that YY1 is able to bind BAP1.

Retinoblastoma-binding protein 7 (RBBP7), a ubiquitously expressed nuclear protein and a component in many histone deacetylase (HDAC) complexes, plays a key role in chaperoning chromatin remodeling proteins to their nuclear histone substrates, including histone acetylases and HDACs [27,28]. RBBP7 modulates the epigenetic activity of these chromatin-remodeling proteins, thereby modulating the expression of target genes [28]. RBBP7 can specifically bind to the BRCT domain of BRCA1 and modulate its transcriptional activity to influence the regulation of cell proliferation and differentiation [29], and have been implicated in numerous cancers [30]. Mass spectrum analysis of human kidney cell line 786-O indicated that BAP1 can interact with RBBP7 [31]. Herein, our results indicate that RBBP7 can bind to BAP1.

Interaction between proteins plays a key role in cell regulation and signaling. Previous studies indicated that BAP1 might inhibit cancer cell growth by interacting with one or more partner proteins, including YY1, RBBP7, HCF-1, H2A, ASXL1/2, and FoxK1/K2 [19,21,25]. YY1 and RBBP7 are the two most important interactive partners of BAP1 [19,31]. By co-immunoprecipitating, we herein further demonstrated that both YY1 and RBBP7 can bind to BAP1 in ccRCC cell line 786-O.

To explore the downstream target proteins of BAP1 in ccRCC, we conducted differential gene expression analysis by knocking down BAP1 in 786-O cells. We identified 3,422 differential expression genes, and a gene cluster was related to 336 pathways. Among them, there is a significant enrichment of differentially expressed genes in pathways such as cycle, senescence, in cancer, Epstein–Barr virus infection (Figure 5, Table 1). Based on differential fold and function association, we selected six differential expression genes, including *E2F*8 (cell cycle), *FGF21* (metabolism)*, HIST1H1B* (nucleosome formation)*, KLF15* (gene transcription)*, HSPA46* (cell proliferation, stress response), and *IL1B* (cell proliferation, inflammation) for further validation. Our qRT-PCR indicated that *E2F*8, *FGF*21, and *HIST1H1B* were down-regulated while *HSPA6* was up-regulated in BAP1-shRNA knockdown 786-o cells, which is consistent with our finding in differential gene expression analysis (Table 3). However, we failed to validate the expression change of *KLF*15 and *IL1B.* The reason is unclear. These results revealed that BAP1 regulates the expression of *E2F*8, *FGF*21, *HIST1H1B,* and *HSPA6* in 786-O cells, which may be associated with tumorigenesis of ccRCC*. E2F*8 is a new member of the E2F gene family and plays a role in modulating the progression of the G1-S phase in the cell cycle. *E2F8* is linked to carcinogenesis [32,33,34]. *FGF*21 (fibroblast growth factor 21), a member of the *FGF* family, is a non-mitosis-promoting gene [35,36,37]. Since ccRCC is a type of metabolic disease. FGF21 is mainly metabolized in the kidney. *HIST1H1B* (histone cluster 1 H1 family member b) encodes a histone that is involved in nucleosome formation [38]. *HIST1H1B* plays a crucial role in the progression of carcinogenesis [38]. *HSPA*6 (Heat Shock Protein Family A [Hsp70] Member 6) is associated with folding, stabilizing, and transportation of proteins and is a member of the stress response pathway. Aberrant expression of HSPA6 is involved in tumorigenesis [39,40]. Based on these results, BAP1 may inhibit carcinogenesis by modulating the cell cycle and metabolism and involving nucleosome formation in ccRCC.

In summary, BAP1 can exert its inhibitory effect on ccRCC by regulating multiple target genes and signaling pathways via interactions with the transcription factor YY1 and the histone-binding protein RBBP7. These findings may have future implications for therapy in BAP1-mutated cancers.
